# Data for spatio-temporal modelling and optimisation of multi-product rice value chains

**DOI:** 10.1016/j.dib.2020.106694

**Published:** 2020-12-24

**Authors:** Stephen S. Doliente, Sheila Samsatli

**Affiliations:** a Department of Chemical Engineering, University of Bath, Claverton Down, Bath BA2 7AY, United Kingdom; b Department of Chemical Engineering, College of Engineering and Agro-Industrial Technology, University of the Philippines Los Baños, College, Los Baños, Laguna 4031, Philippines

**Keywords:** Multi-product rice value chains, Paddy rice yield, Rice farming costs, Cost of rice processing and transport, GHG emissions, Food and non-food demands, Spatio-temporal modelling and optimisation, Value Web Model

## Abstract

A current and fully-referenced dataset of resources and technologies for rice provision system is presented in this paper. These data served as model input data for the first multi-objective spatio-temporal optimisation of Philippine rice value chains. Data on available farmland area and their characteristics, such as paddy rice yield, rice farming costs and GHG emissions, are reported. As scenarios were developed for optimal rice value chains of integrated food and non-food production, estimates on the spatio-temporal demands on food, energy, fuels and chemical are presented. Data on sale prices and GHG emission factors of the raw materials and products are also compiled. Processing and transporting technologies involved in the modelling have their economic and operating parameters presented in this paper. This dataset has been collated through academic journals, technical papers and government agencies; all of which have been properly referenced. These data are valuable to various stakeholders of the rice industry across the globe aiming to understand rice value chains optimisation studies and to conduct further scenario development under different conditions and assumptions.

## Specifications Table

**Table:** 

Subject	Agricultural Economics
Specific subject area	Modelling and optimisation of rice value chains
Type of data	Table
How data were acquired	Data were acquired from academic journals, technical papers and government agencies. All of which are presented and fully referenced in the Supplementary Material (MS Excel format).
Data format	Raw
Parameters for data collection	Realistic national-level estimates for the demands for various resources from rice crop are collected in order to model and optimise multi-product rice value chains for integrated production of food, energy, fuels and chemicals. Reliable estimates for the yields, efficiency, costs and GHG emissions were collected in order to model the economic and environmental impacts of relevant resources and technologies. The rice value chain optimisation model, called the Value Web Model, requires that these input data are in a specific format.
Description of data collection	Data were collected by literature search in academic journals, technical papers and government agencies. Where applicable, the data were processed into a format suitable for input into the rice value chain optimisation model. This article discusses the details of the data processing conducted.
Data source location	The data relate to the rice value chains in the Philippines. Primary sources of data on resources are Philippine government agencies such as the Philippine Statistics Authority [1] and the Philippine Department of Energy [2]. Primary sources of data for processing and transporting technologies are various academic journals and technical papers, which the list of references is provided in the Supplementary Material. Many of these data are applicable to many rice producing countries desiring to understand and optimise their value chains.
Data accessibility	Supplementary Material of this data article, which is also available for download in Mendeley Data via http://dx.doi.org/10.17632/htgr7k5b79.1
Related research article	S.S. Doliente, S. Samsatli, Integrated production of food, energy, fuels and chemicals from rice crops: Multi-objective optimisation for efficient and sustainable value chains, Journal of Cleaner Production, 124900, 2020, https://doi.org/10.1016/j.jclepro.2020.124900. [3]

## Value of the Data

• This article compiles the latest and fully referenced data on resources and technologies required for the first multi-objective spatio-temporal optimisation of rice value chains in the Philippines.
• These data will be useful to investigators seeking to maximise the benefits and minimise the negative impacts of Philippine rice value chains, and other groups needing data on resources and technologies within rice value chains.
• These data have been used for the development of optimal scenarios for integrated food and non-food production of the Philippine rice value chains, which researchers in this subject area can utilise for further investigation and cross-checking of assumptions with other models and/or rice producing nations.
• Publishing these data provides a template in processing and organising the resource and technology data of many rice-producing countries for their appropriate use in spatio-temporal tools and techniques in aid of complex decision-making.

## 1 Data Description

This data article presents the compilation of model input data for the first multi-objective spatio-temporal optimisation of rice value chains in the Philippines. The data were utilised for the scenario development of rice value chains for optimal food (white rice) production only, optimal integrated production of food (white rice) and energy (bio-electricity), and optimal integrated production of food (white rice), energy (bio-electricity), fuels (bio-FT diesel, bio-FT jet and bioethanol) and chemicals (bio-naphtha). For the mathematical formulation of the multi-objective spatio-temporal mixed-integer linear programming model based on the Value Web Model [4], [5], [6], scenarios developed, and insights drawn of the modelling and optimisation of the Philippine rice value chains, readers are directed to this work [3]. All model input data are compiled in the Supplementary Material of this data article, which includes the following:

• Total land area and farmland area in each provinces or zones of the Philippines;
• Farmland characteristics such as paddy rice yield, rice farming costs, and rice farming GHG emissions;
• Spatio-temporal demands on food, energy, fuels and chemicals;
• Sale price and GHG emission factor of resources;
• Cost and operational data of various processing technologies (e.g. rice mill, boiler-steam turbine) and transporting technologies (e.g. truck and barges).

The Supplementary Material provides detailed description of these data. Section 2 discusses further details of data processing needed.

## 2 Experimental Design, Materials and Methods

### 2.1 Spatial representation

Spatio-temporal data presented in this article must be referred to the spatial representation of Philippines depicting 81 provinces in the related research article [3]. Each province or zone has a coordinate $\left(x_{z},y_{z}\right)$ to represent its demand centre. These demand centres were processed in ArcMap (ArcGIS Desktop 10.5 (advanced license), version 10.5.0.6491) using population density data from WorldPop [7] and provincial boundaries from OCHA Philippines [8].

### 2.2 Calculation of the farmland characteristics in the Philippines

The following data were collected from the Philippine Statistics Authority [1]: 1) harvested paddy rice of each province in odt/yr; 2) harvested area of each province in ha; 3) paddy rice production cost based on area of each province in PhP/ha/yr; 4) total land area of each province in ha; and 5) farmland (or arable land) area of each province in ha. By dividing harvested paddy rice of each province with harvested area of each province, the paddy rice yield potential of each province in odt/ha/yr was determined. The quantity of rice straw of each province in odt/ha/yr was estimated from paddy rice yield potential by multiplying with 0.75 residue-to-crop ratio and adjusted with 15% for the quantity of rice straw set aside for soil nutrients replenishment and other applications in the Philippines [9,10]. By dividing paddy rice production cost based on area of each province with paddy rice yield potential of each province, the cost of paddy rice production of each province in PhP/odt was computed. By dividing country average rice cultivation GHG emission factor of 3.11 × 10^3^ kgCO_2_e/ha [11] with paddy rice yield potential of each province, the GHG emissions of paddy rice production of each province in kgCO_2_e/odt was calculated. Tables 1 and 2 of the Supplementary Material tabulate the available farmlands and farmland characteristics per province, respectively.

### 2.3 Calculation of the hourly demands for food, energy, fuels and chemicals

Annual consumption data on white rice was collected from the Philippine Statistics Authority [1]; while annual consumption data on grid electricity, diesel, jet fuel and naphtha were obtained from the Philippine Department of Energy [2]. Annual consumption data on bioethanol was provided by the USDA Foreign Agricultural Service [12]. Population data of each province, also collected from the Philippine Statistics Authority [1], rendered the spatial demand variation across the provinces. Table 3 of the Supplementary Material tabulates the estimated hourly demands for food, energy, fuels and chemicals per province.

### 2.4 Estimation of the properties of resources

The properties of the resources (primary, intermediates and products) of the rice value chains were collated from various sources. Table 4 of the Supplementary Material provides data (when applicable to the case studies) on the sale prices and GHG emission factors.

### 2.5 Estimation of characteristics and conversion efficiencies of processing technologies

Tables 5 and 6 of the Supplementary Material show the estimated data of the characteristics and conversion efficiencies for the processing technologies, respectively. An assumption of 5% of the capital economic impact of a processing technology was used to estimate its fixed O&M economic impact. The capital environmental impact of each processing technology was estimated by multiplying its typical land footprint (in m^2^) with an average associated GHG emissions of constructing per unit area of 1665 kgCO_2_e/m^2^ [13]. An assumption of 5% of the capital environmental impact was also used to estimate the fixed O&M environmental impact.

### 2.6 Estimation of characteristics and conversion efficiencies of transporting technologies

The last three tables in the Supplementary Material present the estimated data on the characteristics (Table 7) and conversion efficiencies (Tables 8 and 9) of the transporting technologies and their infrastructures. Physical losses during resource transport are due to the inefficient existing Philippine transport and logistics system [14,15]. However, the losses occurring at the destination were assumed more significant compared to the losses per transport distance due to unavailability of storage facilities in the country [15]. Thus, distance-independent losses only were considered. In Table 8 of the Supplementary Material, transport efficiency X at the destination was varied at values 0.60, 0.70. 0.80, 0.90, 0.95 and 1.00 in order to study the effect of transport losses on rice value chains for food production only. In Table 9 of the Supplementary Material, transport efficiency at the destination was set to 0.80 for rice value chains with integrated food and non-food production in order to embody the inefficiencies of existing Philippine freight transport system [15].

## Declaration of Competing Interest

The authors declare that they have no known competing financial interests or personal relationships which have, or could be perceived to have, influenced the work reported in this article.

## Appendix Supplementary materials

Image, application 1

## Footnotes

Supplementary material associated with this article can be found in the online version at doi:10.1016/j.dib.2020.106694.
